# Supplementary material for: MicroRNA-199a-5p attenuates blood-brain barrier disruption following ischemic stroke by regulating PI3K/Akt signaling pathway
Source: PLoS One. 2024 Sep 20;19(9):e0306793. doi: 10.1371/journal.pone.0306793 (PMC11414938; doi:10.1371/journal.pone.0306793)
Supplement: Attachment — Submitted filename: PONE-D-24-00772.docx [file pone.0306793.s001.docx]

In this manuscript, the authors studied the preventive and therapeutic effects of miR-199a-5p through PI3K/Akt pathway on ischemia stroke. On this basis, they further investigated the role of miR-199a-5p in BBB. Despite some highlights, this paper has a number of shortcomings, such as such as the logic of manuscript, the data etc, and page numbers and line numbers are missing.

1. The introduction lacks logic. Especially, it did not describe the relationship between miRNA and BBB. Please describe in detail.
2. Where are the tables? I can’t find them. Neurological function scores are better presented as bar graphs than table.
3. The improvement effect of miRNA on MCAO was only evaluated with neurological function score. It should add the data of infarct size and pathological status.
4. Although this study provides detailed evidence of the efficacy of CC, it does not provide in-depth mechanistic validation.
5. There is no logic in the results section. It should be showed from effect to mechanism.
6. Fig. 6A should add the merged figure.
7. The discussion lacks logic. Moreover, miR-199a-5p, as the key to this paper, has not been highlighted in the discussion.
8. Another obvious problem with this paper is lack of sufficient explanation of the results. More explanations on them seem necessary and helpful to readers.
9. The first paragraph of the discussion requires a brief summary of the research results.
10. It is noted that your manuscript needs careful editing by someone with expertise in technical English editing paying particular attention to English grammar, spelling, and sentence structure so that the goals and results of the study are clear to the reader.
