# Supplementary material for: MicroRNA-199a-5p attenuates blood-brain barrier disruption following ischemic stroke by regulating PI3K/Akt signaling pathway
Source: PLoS One. 2024 Sep 20;19(9):e0306793. doi: 10.1371/journal.pone.0306793 (PMC11414938; doi:10.1371/journal.pone.0306793)
Supplement: Attachment — Submitted filename: Response to Reviewers.docx [file pone.0306793.s003.docx]

Response to Reviewers

Dear Editors and Reviewers:

Thank you for your letter and for the reviewers’ comments concerning our manuscript entitled “MicroRNA-199a-5p attenuates blood-brain barrier disruption following ischemic stroke by regulating PI3K/Akt signaling pathway”. (ID: PONE-D-24-00772). Those comments are all valuable and very helpful for revising and improving our paper, as well as the important guiding significance to the research. We have studied comments carefully and have made correction which we hope meet with approval. Revised portion are marked in red in the paper. The main corrections in the paper and the responds to the reviewer’s comments are as following:

Despite Reviewer#1 rejecting the manuscript, in my opinion, the authors improved the manuscript substantially. I suggest revisiting it and inserting a separate section with the study limitations (the authors should insert the limitations described by reviewer#1). Please, insert it before conclusion: in this way, it could be published.

Response: The study limitations have been inserted before conclusion.
